# The effect of the elongation of the proximal aorta on the estimation of the aortic wall distensibility

**DOI:** 10.1007/s10237-020-01371-y

**Published:** 2020-07-31

**Authors:** Stamatia Z. Pagoulatou, Mauro Ferraro, Bram Trachet, Vasiliki Bikia, Georgios Rovas, Lindsey A. Crowe, Jean-Paul Vallée, Dionysios Adamopoulos, Nikolaos Stergiopulos

**Affiliations:** 1 Laboratory of Hemodynamics and Cardiovascular Technology (LHTC), IBI-STI, Ecole Polytechnique Fédérale de Lausanne (EPFL), MED 3 2226 (Batiment MED), Station 9, 1015 Lausanne, Switzerland; 2 Biofluid, Tissue and Solid Mechanics for Medical Applications (BIOMMEDA), Institute of Biomedical Technology, Ghent University, Ghent, Belgium; 3 Department of Radiology and Medical Informatics, Hôpitaux Universitaires de Genève (HUG), Geneva, Switzerland; 4 Department of Cardiology, Hôpitaux Universitaires de Genève (HUG), Geneva, Switzerland

**Keywords:** Cross-sectional area compliance, Axial stretch, Proximal aorta, Finite element analysis

## Abstract

The compliance of the proximal aortic wall is a major determinant of cardiac afterload. Aortic compliance is often estimated based on cross-sectional area changes over the pulse pressure, under the assumption of a negligible longitudinal stretch during the pulse. However, the proximal aorta is subjected to significant axial stretch during cardiac contraction. In the present study, we sought to evaluate the importance of axial stretch on compliance estimation by undertaking both an in silico and an in vivo approach. In the computational analysis, we developed a 3-D finite element model of the proximal aorta and investigated the discrepancy between the actual wall compliance to the value estimated after neglecting the longitudinal stretch of the aorta. A parameter sensitivity analysis was further conducted to show how increased material stiffness and increased aortic root motion might amplify the estimation errors (discrepancies between actual and estimated distensibility ranging from − 20 to − 62%). Axial and circumferential aortic deformation during ventricular contraction was also evaluated in vivo based on MR images of the aorta of 3 healthy young volunteers. The in vivo results were in good qualitative agreement with the computational analysis (underestimation errors ranging from − 26 to − 44%, with increased errors reflecting higher aortic root displacement). Both the in silico and in vivo findings suggest that neglecting the longitudinal strain during contraction might lead to severe underestimation of local aortic compliance, particularly in the case of women who tend to have higher aortic root motion or in subjects with stiff aortas.

## Introduction

Arterial compliance is a major determinant of the cardiac afterload and, in consequence, of the pressure and flow resulting from the interaction of the heart with the arterial system. Approximately half of the total arterial compliance resides in the proximal aorta (Ioannou et al. 2003). The ability of the aortic wall to distend during systole serves as a powerful mechanism to limit the increase in blood pressure and to reduce the cardiac afterload (DeLoach and Townsend 2008). Consequently, a decrease in aortic compliance, as a result of aging or pathology, plays an important role in the development of hypertension and is a strong predictor of all-cause mortality (Vlachopoulos et al. 2010). In this context, accurate estimation of the aortic compliance might become important in the future for correct risk stratification and optimized patient management.

Volume compliance ($$C_{\text{v}}$$) is defined as the change in lumen volume over a change in distending pressure, $$C_{\text{v}} = {\raise0.7ex\hbox{${{\text{d}}V}$} \!\mathord{\left/ {\vphantom {{{\text{d}}V} {{\text{d}}P}}}\right.\kern-0pt} \!\lower0.7ex\hbox{${{\text{d}}P}$}}$$. However, direct measurement of regional blood volume during the cardiac cycle is challenging. The common clinical practice is to derive aortic compliance from cine Magnetic Resonance (MR) images taken perpendicular to the aortic centerline, whereby the maximal and minimal lumen cross-sectional areas are calculated. This measure is referred to as the local or cross-sectional area compliance ($$C_{\text{A}}$$), $$C_{\text{A}} = {\raise0.7ex\hbox{${{\text{d}}A}$} \!\mathord{\left/ {\vphantom {{{\text{d}}A} {{\text{d}}P}}}\right.\kern-0pt} \!\lower0.7ex\hbox{${{\text{d}}P}$}}$$, and has been extensively used in the past (Mohiaddin et al. 1989; Resnick et al. 1997; Vulliémoz et al. 2002; Duprez et al. 2007; Soljanlahti et al. 2008; Lalande et al. 2008).

Area compliance is often used to derive volume compliance. The derivation is based on the assumption that the deformation of the vessel takes place primarily in the radial direction and that there is no significant longitudinal stretch during the cardiac cycle. To illustrate this point, we may consider a non-tapered arterial segment. Any change in its volume (*V*) can be expressed as a function of changes in the corresponding area (*A*) and centerline length (*L*) as follows:

$$\delta V = \delta \left( {A*L} \right) = L*\delta A + A*\delta L \cong L*\delta A$$

In this equation, the assumption that the aorta does not change its length during the cardiac cycle is equivalent to assuming that $\delta L \cong 0$. However, previous studies (Bell et al. 2014; Plonek et al. 2018) have questioned this simplification, particularly for the case of the proximal aorta. It has been demonstrated that during systole the heart pulls the proximal aorta toward the left ventricular apex, which stays practically in place. Plonek et al. (2018) studied the axial motion of the aortic annulus in a population comprising both young and old individuals (*n* = 73) and reported significant longitudinal displacement values from diastole to systole, with an average of 11.6 ± 2.9 mm.

These findings inevitably lead to the following question: How important is the contribution of axial elongation to the volume compliance of the proximal aorta? In order to answer this question, one should quantify the elongation of the aortic root during systole and calculate the errors in the estimation of aortic compliance when axial elongation is neglected. To this aim, we adopted both an in silico (Part I) and an in vivo (Part II) approach. The in silico approach involved the development of a computational framework to simulate the three-dimensional (3-D) aortic wall deformation during the cardiac cycle and to compare the actual wall distensibility (imposed as a model input) to the distensibility estimated after neglecting the longitudinal stretch of the aorta during contraction. In Part II, we validated the in silico results in vivo. More specifically, we collected MR data of the proximal aorta of three healthy young adults during diastole and peak systole and examined the axial and circumferential aortic deformation during LV contraction and the impact of neglected axial stretch on the estimated compliance.

## Materials and methods

### Part I: in silico investigation

#### Image data

**Fig. 1:**
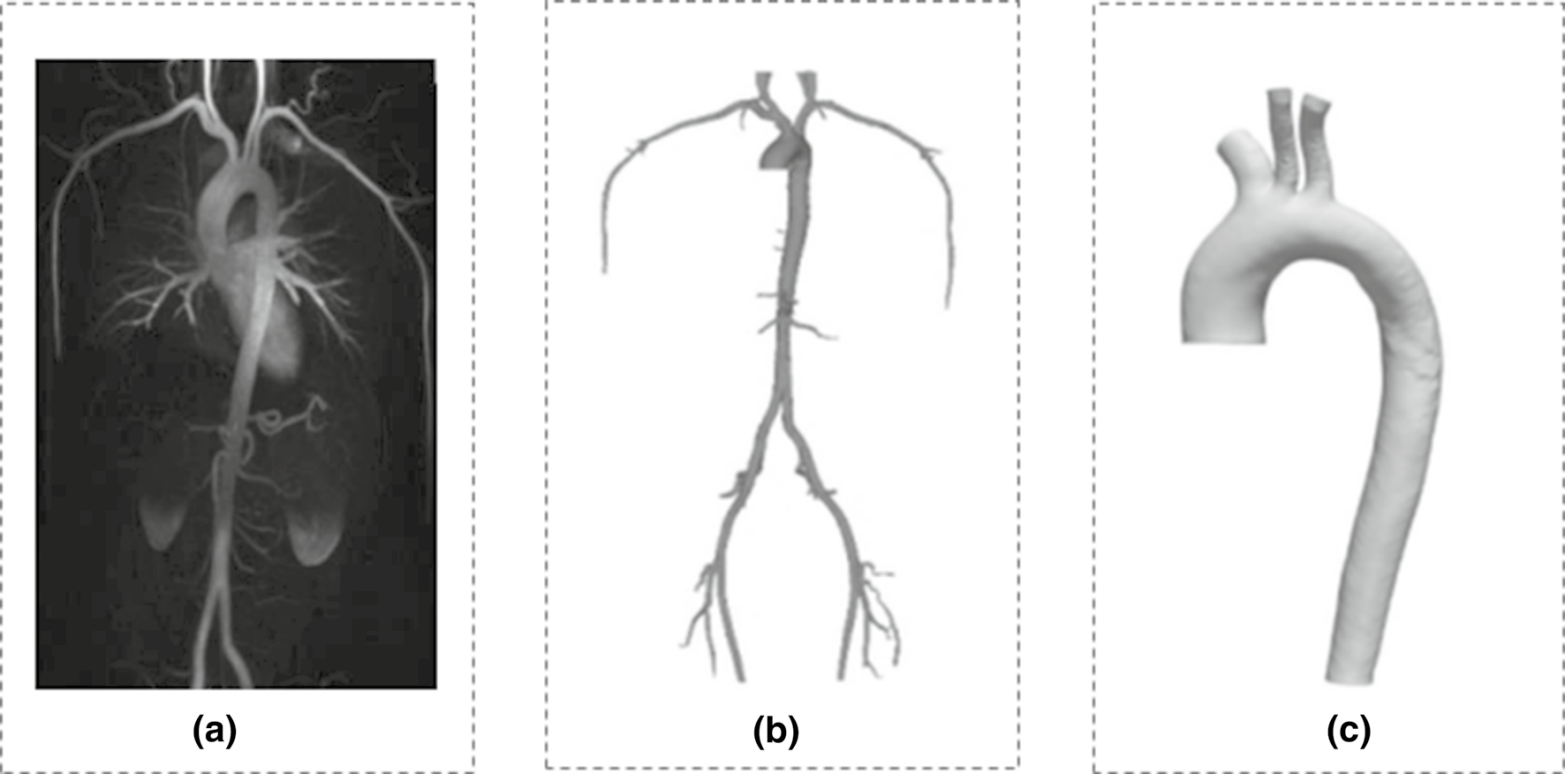
**a** Raw MR data of the aorta and its main branches acquired with ToF MR angiography on a healthy young male (Reymond et al. 2011). **b** Reconstruction of the 3-D geometry. **c** Cropping of the aorta at the main supra-aortic branches and above the celiac trunk

To build the finite element model (FEM) of the aorta, we used the images of the aortic geometry of a healthy 30-year-old male volunteer (height 183 cm, weight 90 kg) acquired with magnetic resonance imaging (MRI) in the context of a previous study (Reymond et al. 2011) (Fig. 1a). The MR angiography (time of flight—ToF, non-ECG-gated) measurement was carried out on a 3T scanner (Siemens Trio-Tim 3T, Germany). The volunteer’s heart rate during this acquisition was 61 bpm. Informed consent was obtained from the subject prior to the scan. Details on the protocol can be found in the original publication by Reymond et al. (2011).

#### Segmentation and mesh generation

As described in Reymond et al. (2011), the segmentation of the aortic geometry was performed from the MR magnitude data following an edge detection method according to the contrast intensity gradient (ITK Snap Software) (Fig. 1b). The final 3-D geometry was cropped to isolate the proximal domain, i.e., starting from the level of the right pulmonary artery and extending down to the celiac trunk (Fig. 1c). The 3-D centerline of this aortic segment was extracted using the open source software Vascular Modeling ToolKit (VMTK) (Antiga et al. 2008).

Subsequently, an unstructured hexahedral mesh was created using the semi-automated algorithm developed by Bols et al. (2016). More specifically, a preliminary multi-block structure was first generated following the branching topology and was then refined. The multi-block structure was projected onto the input surface geometry, and a body-fitted grid was obtained. From the hex core, the boundary layer grid was computed. We assumed that the wall thickness varied along the structure and was 10% of the local lumen diameter. This assumption is often adopted in the literature when no ex vivo data are available (Humphrey et al. 2009). The resulting mesh was composed of 172,533 nodes and 114,568 hybrid hexahedral elements (type C3D8H), with two layers of elements across the vessel wall thickness.

#### Constitutive material model

The constitutive model for the arterial wall was based on Holzapfel et al. (2000) with the extension of Gasser et al. (2006). Hereafter, we will refer to this model as the ‘Holzapfel–Gasser–Ogden’ (HGO) model. The HGO model is a built-in constitutive model in ABAQUS. It assumes that the material is incompressible and consists of an isotropic matrix, wherein $N$ families of collagen fibers are embedded and dispersed around a mean orientation. In this work, we assumed that the material contains $N = 2$ families of fibers, as often assumed in the literature (Roy et al. 2014).

The constitutive model is described by a strain-energy function, $U$, which relates the energy per unit reference volume to strain and stress. The strain-energy function may then be decomposed into a volumetric response, an isochoric isotropic and an isochoric anisotropic response as follows:

1 $$U = U_{\text{vol}} + U_{\text{iso}} + U_{\text{aniso}} = \frac{1}{D}\left( {\frac{{\left( {J^{\text{el}} } \right)^{2} - 1}}{2} - \ln J^{\text{el}} } \right) + C_{10} \left( {\bar{I}_{1} - 3} \right) + \frac{{k_{1} }}{{2k_{2} }}\mathop \sum \limits_{a = 1}^{N = 2} \left\{ {\exp \left[ {k_{2} \bar{E}_{a}^{2} } \right] - 1} \right\}$$

with

2 $$\bar{E}_{1} = \kappa \left( {\bar{I}_{1} - 3} \right) + \left( {1 - 3\kappa } \right)\left( {\bar{I}_{4a} - 1} \right) \,{\text{and}}\, \bar{E}_{2} = \kappa \left( {\bar{I}_{1} - 3} \right) + \left( {1 - 3\kappa } \right)\left( {\bar{I}_{6a} - 1} \right)$$

To approximate the physiological vascular wall response, we chose the material properties according to the population-averaged values proposed in the literature for young male adults. Parameter *D* was set at 10^−6^ kPa, as recommended to ensure incompressibility (Roy et al. 2014). The two families of fibers were assumed symmetrically oriented, making an angle $\alpha = 55^{ \circ }$ with respect to the circumferential direction. This value was chosen based on physiological data (Åstrand 2008; Roccabianca et al. 2014). The dispersion coefficient $\kappa$ was set to a high value, $\kappa = 0.315$, similarly to Roy et al. (2014).

The remaining material parameters,$C_{10}$, $k_{1}$ and $k_{2}$, were approximated according to two criteria: first, the values should be in the physiological range proposed in the literature (Holzapfel et al. 2000; Åstrand 2008; Pasta et al. 2016; Huh et al. 2019), and, second, the deformation of the aortic wall under the pressure load should reflect the expected elasticity of the aortic wall. From uniaxial tension tests, the three parameters are known to be in the following range: $C_{10} \in \left[ {1, 240} \right]$ kPa, $k_{1} \in \left[ {1,410} \right]$ kPa, $k_{2}$ $\in \left[ {2.5, 72} \right]$. The elasticity of the aortic wall was estimated according to the Bramwell–Hill equation using the aortic pulse wave velocity (PWV) measured in the original study by Reymond et al. (2011), ${\text{PWV}} = 4.8 \,{\text{m}}/{\text{s}}$. To match the measured PWV and the aforementioned literature guidelines, the final material parameters were set to the physiological values $C_{10} = 42\,{\text{kPa}}$, $k_{1} = 290\, {\text{kPa}}$ and $k_{2} = 12.6$.

#### Optimization of fiber orientation

When defining the mean direction of the different families of fibers, one needs to account for the tortuosity of the aortic geometry. In order to define the mean angle direction in a consistent manner throughout the aortic domain, we need to consider a local coordinate system for each finite element. To do so, we developed a MATLAB code in which we adapted the orientation of the collagen fibers following the lumen centerline, similarly to Roy et al. (2014).

#### Zero-pressure geometry

At the time of the scan, the aorta is deformed under physiological pressure. This means that the aortic geometry we obtain after segmentation corresponds to the loaded state. To perform the FEM analysis, we need to define the unloaded configuration. A number of studies in the literature propose inverse problem-solving techniques, whereby the unloaded configuration is calculated from the known in vivo measured geometry and the measured distending pressure. In this work particularly, we follow the fixed-point optimization approach of Bols et al. (2013). The zero-pressure configuration was restored by iteratively updating the coordinates of the unloaded geometry until the deformed geometry at physiological pressure matched closely the in vivo measured configuration. This optimization code was written in MATLAB, and at each optimization cycle, the updated mesh coordinates were communicated to the finite element solver (ABAQUS). For the restoration of the zero-pressure configuration, we assumed that the distending pressure at the time of the measurement was equal to the measured diastolic pressure.

#### Load and boundary conditions

##### Pressure load

The time-varying pressure load acting on the inner aortic wall was assumed equal to the pressure curve measured at the right common carotid artery of the subject the same day as the scan. Carotid pressure waveform was acquired over 10 heart cycles with applanation tonometry (Millar Instruments, SPT 301, Houston, TX, USA) and was calibrated according to the measured mean and diastolic brachial pressures. The systolic (SBP) and diastolic (DBP) blood pressure were 110 mmHg and 70 mmHg, respectively.

##### Viscoelastic external tissue support

**Fig. 2:**
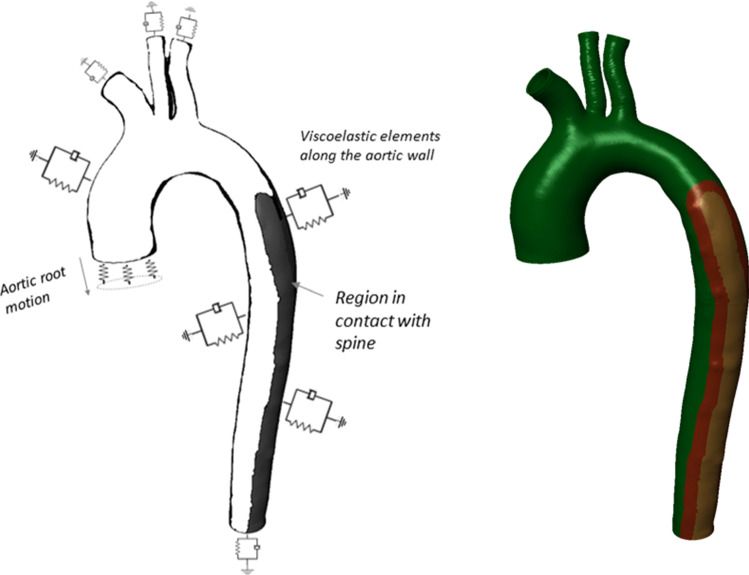
Boundary conditions (left) Schematic representation of the viscoelastic boundary conditions applied along the vessel wall to mimic the support provided by the external tissues and organs. The measured aortic root displacement is prescribed at the proximal end via stiff springs. Adapted from Moireau et al. (2012). (right) Different regions of the wall domain considered for assigning viscoelastic boundary properties. These regions were identified according to the position of the aorta relative to the spine. The orange region is in contact with the spine, the red region is in the spine vicinity, and the green region is less constrained by the surrounding tissues

When applying boundary conditions along the aortic wall, one needs to consider the external support provided by the surrounding tissues. Interestingly, this support is exerted non-uniformly throughout the domain. The spine significantly tethers a part of the descending thoracic aorta, whereas the remaining wall is less constrained. The majority of approaches in the literature neglect this fact and apply a constant external pressure all along the outer surface. As explained by Moireau et al. (2012), this boundary condition results in artificial motion patterns of the arterial wall. Conversely, their work (Moireau et al. 2012) included the viscoelastic, non-uniform support provided by the extremal tissues. In the present study, we therefore adopt their approach. More specifically, we applied along the outer aortic wall the extended Robin boundary condition proposed in (Moireau et al. 2012), which models an elastic and a viscoelastic response of the external tissue. W:

$$\sigma \cdot \underset{\raise0.3em\hbox{$\smash{\scriptscriptstyle-}$}}{n} = - k\underset{\raise0.3em\hbox{$\smash{\scriptscriptstyle-}$}}{y} - c\underset{\raise0.3em\hbox{$\smash{\scriptscriptstyle-}$}}{u} - p_{o} \cdot \underset{\raise0.3em\hbox{$\smash{\scriptscriptstyle-}$}}{n}$$

where $$\sigma_{ }$$ is the Cauchy stress tensor, the terms $$k\underset{\raise0.3em\hbox{$\smash{\scriptscriptstyle-}$}}{y}$$ and $$c\underset{\raise0.3em\hbox{$\smash{\scriptscriptstyle-}$}}{u}$$ represent the elastic and viscoelastic responses, respectively, and $$p_{o}$$ is the intrathoracic pressure. The pressure $$p_{o}$$ can be neglected in our case as the scan was performed during breath hold. We imposed this condition perpendicular to the outer aortic wall by connecting each node of the outer surface of the mesh to a spring of stiffness $$k$$ and a dashpot of damping coefficient $$c$$. The values of $$k$$ and $$c$$ varied according to the position of the node relatively to the spine. To achieve this, we divided the solid mesh into 3 regions: (a) a region in direct contact with spine, (b) in the spine vicinity and (c) opposite to the spine. The region in contact with the spine was identified by locating the intercostal arteries. A schematic representation of these three regions is shown in Fig. 2. For the different regions, the parameters $$k$$ and $$c$$ were chosen according to the values provided by Moireau et al. (2012) for a young adult. Time-varying effects were neglected. The same Robin boundary condition was used for the outlets, with the parameters $$k$$ and $$c$$ assumed equal to the reported values in Moireau et al. (2012) (Fig. 2).

##### Heart motion

To account for the aortic root motion, we prescribed a measured displacement field $y_{b}$ on the nodes of the proximal boundary of the aortic wall (Fig. 2). This coupling was achieved indirectly by imposing the displacement $y_{b}$ on reference points connected to the proximal boundary via stiff springs, as in Moireau et al. (2012). The use of springs was necessary in order to allow for the radial expansion of the aorta at the inlet. Additionally, high quality data of the complex heart motion that would justify the direct enforcement of the displacement $y_{b}$ were not available. The imposed displacement $y_{b}$ was measured from dynamic MR images of the aortic root collected on a different 28-year-old male subject (height 184 cm, weight 79 kg). The MR examination was carried out on a 3T clinical MRI scanner (MAGNETOM Trio, Siemens AG, Healthcare Sector, Erlangen, Germany) in the context of our ongoing research study *(Project ID CER*-*VD 2017*-*00954)*. Approval from the local ethical committee was obtained, and the volunteer gave informed consent prior to inclusion. The measurement was performed under breath hold over 7 heart beats in cine TrueFISP sequences (TR 29.6 ms, TE 1.3 ms, flip angle 30°, resolution 1.4 mm × 1.4 mm × 8 mm). Average heart rate during this acquisition was 63 bpm. The generalized autocalibrating partially parallel acquisition (GRAPPA 3) reconstruction was used.

**Fig. 3:**
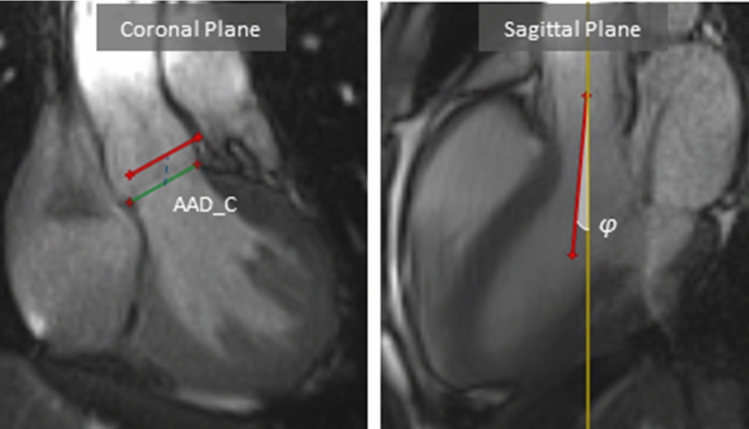
Motion of the aortic root in 2 planes. (left) Coronal cine-MR images showing the position of the ventriculo-aortic junction (VAJ) in systole (green line) and in diastole (red line). The blue dotted line represents the maximal aortic annulus displacement in the coronal plane (AAD_C) linking the mid-points of the VAJ in systole and diastole. (right) Sagittal cine-MRI images depicting the motion vector in the sagittal plane (red arrow) and the cross-reference line (yellow line). The angle, *φ*, between the red vector and yellow line represents the angle of rotation between the two planes. The total displacement from diastole to systole was calculated as AAD_C*cos(*φ*) and was found equal to 9.5 mm

From a visual inspection, the subject presented significant aortic root displacement during the cardiac cycle, a common observation for a young healthy individual. The displacement was calculated at each timeframe by manually tracking the aortic root motion in two planes. First, the position of the ventriculo-aortic junction (VAJ) was established in the coronal plane. The longitudinal displacement of the aortic annulus in this plane was measured between consecutive images as the distance between the mid-points of the VAJ. The estimated displacement was subsequently projected onto the motion vector in the sagittal plane. The methodology is presented in Fig. 3. To account for intra-observer variability, the calculation of the aortic root displacement was conducted again 1 week after the initial assessment. The systolic aortic root displacement was found equal to 9.5 mm (average of two measurements, 9.8 mm and 9.1 mm).

#### Effect of elongation on the estimated distensibility

First, the fixed-point optimization code was run to restore the unloaded configuration. The unloaded geometry was then imported into ABAQUS and inflated to the measured diastolic pressure. Subsequently, we run the complete model with the boundary and loading conditions described above and simulated a full cardiac cycle.

The simulation results were post-processed using VMTK (Antiga et al. 2008), ParaView (Ahrens et al. 2005) and in-house codes. More specifically, the dynamic volume of the domain was extracted for each time increment and the peak systolic (*V*_max_) and diastolic (*V*_min_) values were obtained. The ratio of volume changes over pressure changes was calculated and normalized by the diastolic volume value to yield aortic distensibility, (*V*_max_ − *V*_min_)/*V*_min_/(pulse pressure). This value served as the reference wall distensibility (imposed by the model properties).

Consequently, seven cross sections perpendicular to the centerline were tagged along the aorta and were used to calculate the respective area compliances. We then estimated anew the aortic distensibility by integrating the area compliances over the centerline of the aortic segment (computed automatically using VMTK (Antiga et al. 2008)). For the integration, we used the volume formula for a conical cylinder:

$${\text{Volume}}\, {\text{of }}\,{\text{conical}}\, {\text{cylinder}} = \frac{\pi L}{12}\left( {D_{1}^{2} + D_{1} D_{2} + D_{2}^{2} } \right)$$

where $L$ is the centerline length, $D_{1}$ is the diameter of the proximal cross section, and $D_{2}$ is the diameter of the distal cross section. To assess the effect of neglecting the elongation, we assumed that the length of the aortic segment did not vary during the cardiac cycle and remained equal to its diastolic value. This estimate of distensibility was compared to the reference value.

#### Parametric analysis

The original model parameters were set according to (a) measurements on healthy young adults, (b) literature data pertaining to healthy young males. To account for the generic nature of the model, we performed a sensitivity analysis and examined the effect of two key model parameters on the estimation of distensibility. The first parameter that was varied is the aortic wall compliance. The original material parameters corresponded to the highly compliant aorta of a young subject where the pulse wave theoretically propagates with a velocity of 4.8 m/s. Two additional levels of compliance were simulated, a highly stiff and an intermediate level, while the aortic root motion was kept constant at 9.5 mm. As representative of older individuals, the scenario of a stiff aorta was built based on the evolution of the material properties with aging (Mohiaddin et al. 1989; Moireau et al. 2012). For this combination of parameters, the theoretical PWV was 9 m/s. The intermediate level of compliance was simulated also based on (Åstrand 2008; Pasta et al. 2016), corresponding to a theoretical PWV of approximately 7 m/s.

The second parameter that was investigated is the aortic root motion imposed proximally to the domain. The original displacement profile was measured in a healthy young male as a function of time, the maximal displacement being equal to 9.5 mm. Three additional aortic root motion scenarios were considered: (a) no systolic displacement during the cardiac cycle, (b) a lower systolic displacement of 5 mm and (c) a greater systolic displacement of 15 mm. These values are in the physiological range reported by Plonek et al. (2018) (range of 3–19 mm in a population of 73 young and old adults, with an average of 11.2 ± 2.9 mm).

### Part II: in vivo investigation

#### Subjects

For the in vivo validation, three healthy young subjects were recruited: one 38-year-old male (height: 172 cm, weight: 62 kg) and two 18-year-old females (height: 167 cm, weight: 50 kg, and height: 176 cm, weight: 67 kg). All three subjects were free of any cardiovascular disease, normotensive and non-smokers. The subjects gave written consent prior to inclusion in the study *(Project ID CER*-*VD 2017*-*00954)* and were instructed not to consume any caffeine or food at least 4 h before the measurement.

#### MRI protocol

Non-contrast enhanced MR angiography acquisitions were performed to capture the aortic geometry at diastole and peak systole. The examination was carried out on a 3T clinical MRI scanner (MAGNETOM Trio, Siemens AG, Healthcare Sector, Erlangen, Germany) using spine and body surface coil elements. Oblique sagittal images of the aorta were acquired with 3-D gradient echo sequences (TR 158.22 ms, TE 1.33 ms, flip angle 12°, resolution 0.625 mm × 0.625 mm × 2 mm (32 slices), generalized autocalibrating partially parallel acquisitions (GRAPPA) acceleration factor 2). The trigger delay was set after acquiring cine 2-chamber cardiac MR images and observing the static point in systole or diastole. The window of acquisition was 102 ms. Respiratory navigation with around 50% acceptance window was used to reduce respiratory motion artifacts. The acquisition time was approximately 3 min.

#### Data processing and analysis

**Fig. 4:**
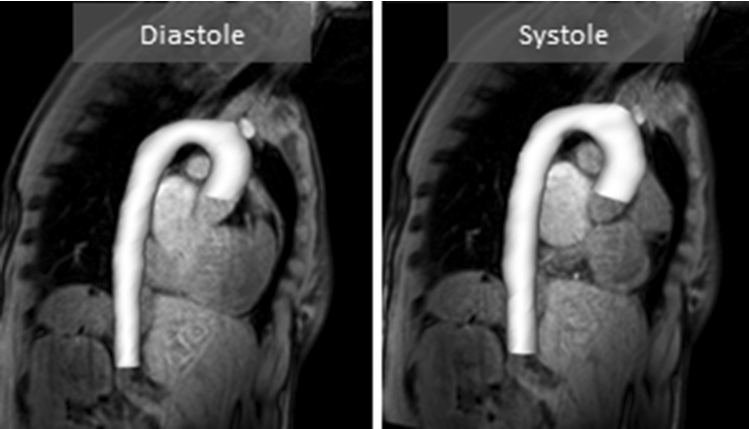
Imaging of the proximal aorta of the 38-year-old male during diastole (left) and systole (right) in the sagittal plane along with the segmented 3-D geometries

Initially, the systolic longitudinal displacement of the aortic root was estimated in the sagittal plane by tracking the right coronary artery at systole and diastole. Subsequently, for each subject, the proximal aortic geometry was extracted from the MR angiography data in the peak systolic and diastolic timeframes using the open source software 3D Slicer (Kikinis et al. 2014). The final configuration for peak systole and diastole included the vascular lumen of the ascending and descending aorta, extending down to the celiac trunk. The major neck vessels were truncated (Fig. 4). After acquiring the 3-D configurations, the volume of the aortic lumen was extracted at peak systole and diastole using VMTK (Antiga et al. 2008). The reference aortic wall distensibility was calculated as (*V*_max_ − *V*_min_)/*V*_min_/(pulse pressure).

Subsequently, eight characteristic cross sections along the aorta were tagged: the proximal and distal end, before the brachiocephalic artery, before the left common carotid artery, before and after the left subclavian artery and at the level of the 1st and 7th intercostal arteries. The area compliance of these cross sections was calculated and aortic distensibility was anew estimated by integration over an invariant diastolic centerline length.

## Results

### Part I: in silico Investigation

#### Zero-pressure configuration

**Fig. 5:**
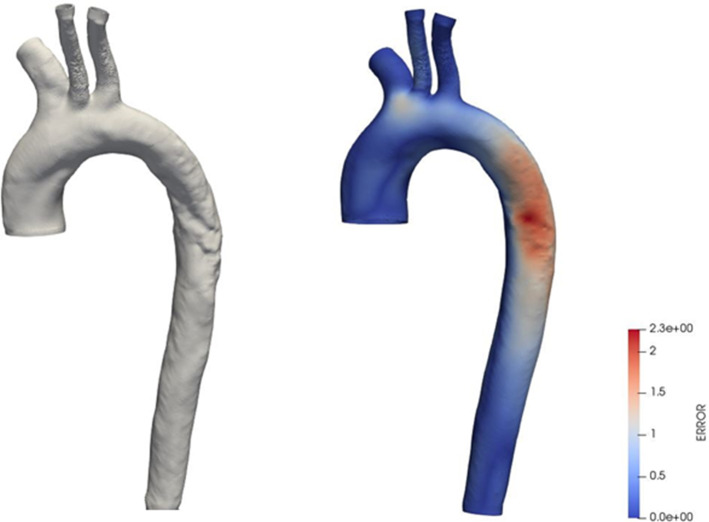
(left) Unloaded configuration after 6 cycles of the fixed-point optimization algorithm (Bols et al. 2013). (right) The corresponding error map between the measured geometry and the configuration inflated to diastolic pressure. Created in ParaView (Ahrens et al. 2005). The error scale is in mm

The aortic geometry was brought to its zero-pressure state, assuming that the internal pressure load was 70 mmHg at the moment of the scan (Fig. 5). The optimization algorithm was terminated by the user after 6 cycles, because for a higher number of iterations the structure presented buckling close to the brachiocephalic bifurcation and the algorithm diverged. After 6 simulations, the maximal error was in the order of magnitude of 8% of the local arterial diameter. Figure 5 also depicts the error map between the measured geometry and the optimized configuration inflated to diastolic pressure.

#### Reference distensibility vs distensibility estimate in absence of axial stretch

##### Generic young model

**Fig. 6:**
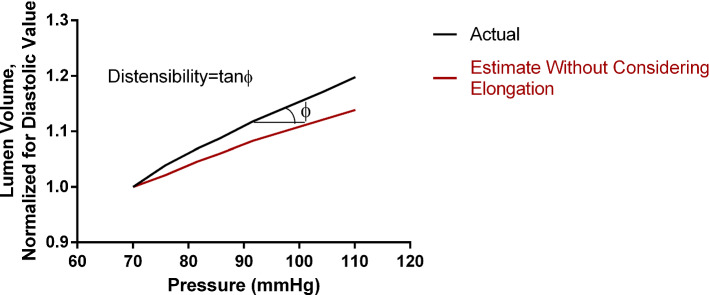
Generic model results for the lumen volumetric changes with increasing pressure. Comparison between the actual volumes (black line) against the estimates after neglecting (red line) the effect of elongation. The aortic distensibility is equal to the slope of the curve

Figure 6 shows the model-derived lumen volumetric changes with increasing pressure. The black curve represents the actual volume of the 3-D lumen, and the red curve the estimation after neglecting the elongation of the ascending aorta. The volumes are normalized to the volume at diastolic pressure. The slope of each curve represents the aortic distensibility. We clearly note a significant underestimation of the aortic wall distensibility when the longitudinal stretch is not accounted for (error of 30.2%).

##### Sensitivity analysis

Table 1 summarizes the simulation results for the generic case as well as after varying two key model parameters, i.e., the wall compliance and the imposed aortic root motion. The table compares the distensibility values as calculated by the actual volume changes (reference) against the estimates after neglecting the longitudinal stretch. Three levels of compliance are examined, from highly compliant to stiff. The corresponding PWVs range from 4.8 to 9 m/s. Note that the same level of aortic root displacement was imposed while varying the wall material properties. We observe that as the stiffness of the wall increases, the underestimation of wall elasticity rises, namely the error doubles from − 30.2% in the compliant wall case to − 61.9% in the stiff wall case. Furthermore, four levels of aortic root motion are presented. As expected, greater aortic root displacements—and thus elongation—lead to more pronounced underestimation, from − 20.5% in the no motion scenario to − 36.7% in the significant displacement scenario.

**Table 1:** Simulation results after varying key model parameters

	Reference distensibility (10^−3^ mmHg^−1^)	Distensibility estimate without considering elongation (10^−3^ mmHg^−1^)	Error without considering elongation (%)
Generic young model (PWV = 4.8 m/s and with aortic root displacement of 9.5 mm)	4.94	3.45	− 30.2
Parameter sensitivity			
Compliance			
Intermediate level (theoretical PWV = 7 m/s)	3.01	1.86	− 41.2
Stiff (theoretical PWV = 9 m/s)	1.55	0.61	− 61.9
Aortic root motion			
No displacement	4.45	3.59	− 20.5
Displacement of 5 mm	4.66	3.54	− 24.1
Displacement of 15 mm	5.25	3.33	− 36.7

The table includes the reference distensibility value along with the estimate without considering elongation. The respective errors are shown

### Part II: in vivo investigation

Table 2 summarizes the demographic characteristics of the participants as well as the measured aortic root longitudinal displacement. The displacement values were higher for the two young female subjects (15.6 mm and 11.4 mm) when compared to the value for the 38-year-old male subject (8.5 mm). This result is consistent with the literature, which has reported a statistically significant negative correlation between the longitudinal displacement of the aortic annulus and the age of a patient (Plonek et al. 2018). The table also includes the analysis results on the subjects’ distensibility estimation. The estimation errors varied from − 26 to − 44%. Similarly to the in silico results, we observe a tendency for the error to increase with greater aortic root motion. On the other hand, the error is significantly smaller for the older male subject, who has a slightly stiffer aorta. Note that the calculated errors are of comparable magnitude for both the in vivo and the in silico analyses.

**Table 2:** Participant demographic characteristics, measured aortic root displacement and distensibility estimation

Subject	Gender	Age	Height (cm)/weight (kg)	Aortic root displacement (mm)	Reference distensibility (10^−3^ mmHg^−1^)	Distensibility estimate without considering elongation (10^−3^ mmHg^−1^)	Error without considering elongation (%)
1	M	38	172/62	8.5	6.66	4.84	− 25.9
2	F	18	168/50	11.4	7.59	5.13	− 32.5
3	F	18	176/67	15.6	6.73	3.75	− 44.2

## Discussion

This study aimed at questioning the established methodology for the estimation of aortic compliance or distensibility from cross-sectional radial deformations. In the past, a plethora of studies has used cross-sectional area measurements in order to provide insights into the effects of aging (Mohiaddin et al. 1989; Duprez et al. 2007), training (Mohiaddin et al. 1989) and different pathologies (Mohiaddin et al. 1989; Resnick et al. 1997; Soljanlahti et al. 2008; Lalande et al. 2008) on the ascending aortic distensibility. A key argument in favor of neglecting the axial vessel stretch is based on the findings of Patel et al. (1963). In their study, Patel et al. (1963) analyzed aortic pressure and radius in the living dog and found that the ratio of pressure changes to radius changes along the aorta correlated well with measures of impedance (*r* = 0.99). However, it should be stressed that their published data pertain to the descending thoracic aorta, which is not subjected to as significant longitudinal strains when compared to the ascending aorta (Morrison et al. 2009). An approximation that is sufficient for the study of the elasticity of the descending aorta does not necessarily apply to the ascending counterpart.

In fact, the presence of significant ascending aortic longitudinal strain has been noted in several previous observations (Bell et al. 2014; Plonek et al. 2018). An in vivo study by Bell et al. (2014) measured the longitudinal and circumferential strain of the proximal aorta in older adults of both genders and examined the correlation of these values with the measured central pulse wave velocities (which served as the reference for aortic wall elasticity). They found that the central PWV correlated poorly with uncorrected proximal aortic circumferential strain, whereas it was inversely related to longitudinal strain. Longitudinal strain was also associated to other risk factors for higher aortic stiffness such as untreated hypertension. Furthermore, an ex vivo study by Bergel (1961) showed that longitudinal strain affects the shape and value of the aortic pressure–volume curve and suggested that the longitudinal deformation should be accounted for when measuring arterial compliance in vivo.

This work supplements and expands on previous studies by undertaking both a computational and an experimental investigation. Our model-derived results clearly point to a significant underestimation of the aortic wall distensibility when the elongation of the aortic centerline is neglected. Importantly, we observed that the underestimation is not systematically consistent; namely, the errors ranged from − 20 to − 62% according to two key parameters. The first one was the elasticity of the aortic wall. We found that greater stiffness of the aortic wall leads to more pronounced underestimation of the distensibility. This has also been suggested in the past (Bell et al. 2014) and is likely linked to the smaller circumferential strains of older subjects. In other words, the stiffer the wall is circumferentially, the more its compliance will be affected by axial displacements. Moreover, as can be observed in the second parameter study, the effect of the aortic root displacement is non-negligible. For a higher longitudinal displacement of the aortic annulus, we showed that the errors increase substantially. This finding is rather intuitive: when the aortic root is subjected to a higher displacement due to the cardiac contraction, the aorta is more longitudinally stretched and thus is subjected to a smaller radial extension.

Our in silico results compared well with the in vivo acquired data. For all three subjects, the underestimation errors were in the anticipated order of magnitude, from 26 to 44%. Despite the small sample size, we were able to observe an interesting difference in the longitudinal aortic root displacement between the male and the female subjects, which is consistent with the literature. Similarly, we noted a higher underestimation of the aortic distensibility for the two female subjects. Bell et al. (2014) also reported similar gender patterns, as they showed that longitudinally corrected circumferential strains were greater in women in comparison with men (the female average was 14.4% with a range of [13.6, 15.2]%, while the male average was 13.0% with a range of [12.4, 13.7]%, *p* value = 0.01). This is due to the combined effect of a greater aortic root motion and shorter ascending aortas. We expect that the overestimation of aortic stiffness when neglecting the effect of longitudinal strain might therefore be greater in women than in men.

When examining the effect of aging on the underestimation of local distensibility, one needs to consider two different compensatory mechanisms: older subjects have in general stiffer aortas (which should lead to higher underestimation errors) but also exhibit smaller axial displacements (which should lead to smaller underestimation errors). It is not evident, therefore, which of the two mechanisms dominates in a specific patient. In our future work, we plan to study a larger population in order to propose correction models to disentangle the two effects.

An important implication of our findings is that the longitudinal deformation of the proximal aorta might be a parameter of clinical interest. The inclusion of the aortic root longitudinal strain in risk stratification has also been evoked in the past. A recent study by Guala et al. (2019) reported the predictive value of aortic root longitudinal strain for aortic dilation and aortic events in Marfan syndrome patients, while several other studies have demonstrated the potential for aortic longitudinal deformation in the assessment of risk of dissection (Beller et al. 2004; Singh et al. 2016).

### Considerations on the analysis: actual distensibility vs distensibility without considering elongation

In our analysis, we investigated the effect of longitudinal strain by comparing two distensibility values: one obtained from the volumetric deformation of the aorta (reference) and one obtained via integration of the area compliance over an invariant centerline length. Naturally, a part of the comparison errors stems from the integration process itself. That is because the proximal aorta is not a perfectly conical cylinder, and therefore, the integration of the area compliances over the centerline length using the volume formula for a conical cylinder will involve numerical errors. To quantify these numerical errors, we also integrated the area compliances over a variable centerline length, thus accounting for the effect of elongation. Theoretically, the reference wall distensibility and the estimate after accounting for the elongation should perfectly agree. In all in silico and in vivo cases, the stiffness underestimation errors were improved by at least 50% after accounting for the elongation. Concretely, the errors due to the integration were non-negligible, but compared to the results obtained without considering elongation at all they were smaller and consistent. This suggests that, even if the reported differences between actual distensibility and estimation without considering elongation may be exaggerated due to numerical errors, they are certainly not an integration artifact.

### Limitations of the mathematical model

There are two important points regarding our in silico analysis that should be highlighted. Firstly, we adopted a quasi-static approach and hence did not consider wave propagation phenomena. This constitutes a limitation of the study and was done primarily to avoid the expenses of a fluid–structure interaction model. Secondly, the computational model was built from a combination of literature data and in vivo measurements on different individuals, thus reflecting only the generic properties of a healthy young aorta. Indeed, the aim of our study was not to build a precise, state-of-the-art patient-specific model of the aortic wall. Our approach was rather to develop a generic model that captures well the physiological response of the aorta under distending pressure. Accordingly, particular attention was paid to creating a robust hexahedral mesh and implementing physiologically relevant material behaviors and boundary conditions. The generic nature of the model entails certain limitations, particularly if we consider that the literature on the field provides us with rather scarce reference data. To account for that, we conducted a sensitivity analysis and quantified how variations in the model parameters affected our estimations. The results we obtained were physiological and followed the expected patterns.

The longitudinal displacement of the aortic root was manually tracked, which can be subject to observer-dependent errors. To account for the intra-observer variability, the root displacement was measured twice and the average value was used. Additionally, we implemented a high value of the dispersion parameter κ in accordance with previous literature (Roy et al. 2014). For this level of dispersion of the collagen fibers, the material becomes practically isotropic, which might have an impact on the study results. An important parameter that was not examined here is the influence of the geometric configuration, since the entire analysis was performed based on one healthy young aortic geometry. It is known, however, that the aortic geometry differs significantly among individuals of different age, height, gender, etc. The aorta of older subjects is also more tortuous (Redheuil et al. 2011), which might affect its compliance. In the future, we plan to include this parameter and develop multiple models of different aortic configurations from young and old subjects of both genders.

In our future work, we plan to use gated MR acquisitions, in order to ensure that the in vivo measured geometry corresponds indeed to the diastolic configuration; this assumption was made in the present study in order to restore the zero-pressure geometry. The zero-pressure geometry was computed based on the fixed-point optimization method proposed by Bols et al. (2013), which is an iterative algorithm. Previous literature (Peirlinck et al. 2018) suggests that iterative algorithms might lack accuracy and robustness when applied to complex material models, such as the HGO model. This was confirmed by our work, indeed after a few iterations the structure presented buckling at the level of the brachiocephalic bifurcation and the optimization had to be manually terminated. The use of a more sophisticated method to restore the zero-pressure geometry, such as the inverse elastostatics method of Peirlinck et al. (2018), might be more appropriate for such a setup. This potential needs further investigation.

Due to the quasi-static nature of the model developed in the present study, the effect of the elongation of the proximal aorta on the regional aortic compliance as assessed via the pulse wave velocity could not be investigated. Aortic PWV is calculated as the time delay between two pressure or flow waveforms in different locations along the aorta. It is therefore expected that the elongation of the aortic root will alter the distance between the measuring locations and potentially affect the PWV. We plan to examine this hypothesis in our future work.

### Limitations of the in vivo analysis

Although the in vivo analysis was conducted on a small number of subjects, the tendencies we observed matched closely what was expected from both the computational model and the literature. A similar analysis in a large cohort study might reveal gender- and age-related differences that cannot be elucidated in the context of the present work.

Furthermore, we need to acknowledge that the absolute values of distensibility presented here are not precise, given that the real aortic pulse pressure was not measured. Indeed, precise measurement of aortic pressure is invasive and practically impossible in the framework of a study on healthy subjects. However, our goal was not to provide the community with reference values of aortic distensibility, an important work that has been undertaken by several studies in the past. Instead, we used the reference pulse pressure values proposed from large cohort studies as representative of each subject’s age and gender. This simplification does not affect the generality of our conclusions, given that pulse pressure is canceled out during the calculation of the underestimation errors.

## Conclusion and future work

Both our computational and experimental findings point to the same direction. We suggest that neglecting the longitudinal strain during contraction might severely hinder the accurate assessment of the distensibility of the proximal aorta. In this context, the established methodology that examines ascending aortic area changes in the cross-sectional plane might lead to severe underestimation of local aortic compliance, particularly in the case of women or older subjects. Following these promising initial results, our future steps will be focused in i) the expansion of the in silico study to include multiple aortic geometries from subjects of different ages and gender, ii) the in vivo investigation of the correlation between volume compliance and cross-sectional area compliance in a larger cohort study.
